# Resistance or pseudo-resistance?

**DOI:** 10.1192/j.eurpsy.2022.941

**Published:** 2022-09-01

**Authors:** R. Zanardi, F. Attanasio, C. De Cesare, V. Fazio, C. Colombo

**Affiliations:** 1 IRCCS San Raffaele Scientific Institute, Psychiatry - Mood Disorders, Milano, Italy; 2 Università Vita-Salute San Raffaele, Psychiatry, Milano, Italy

**Keywords:** bipolar disorder, pseudo-resistance, depressive disorder, treatment resistant depression

## Abstract

**Introduction:**

Treatment-Resistant Depression continues to represent a great challenge for clinicians.

**Objectives:**

We investigated patients with history of resistance, assessing prognostic factors, response to treatments, and remission over time.

**Methods:**

We recruited 202 unipolar and bipolar depressed inpatients. According to anamnestic backgrounds, patients were assigned to: A) *Non-resistant *: responders, with no characteristics of resistance in the current episode. B) *Resistant*: resistant to two antidepressant trials of adequate doses and duration. C) *Pseudo-resistant *: non-responders, not classifiable as *Resistant* because of inadequate trials. During hospitalization, patients were treated by clinical judgment, following a rehabilitation program.

**Results:**

**Figure:**
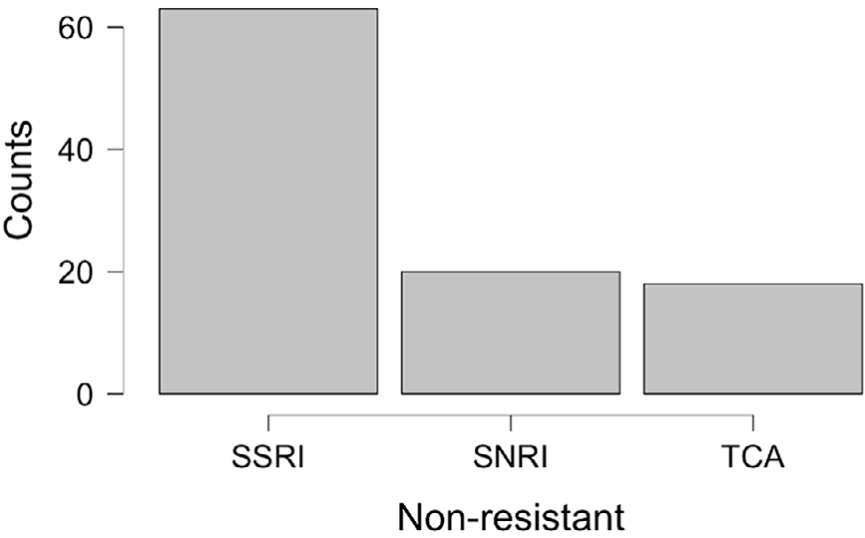

**Figure:**
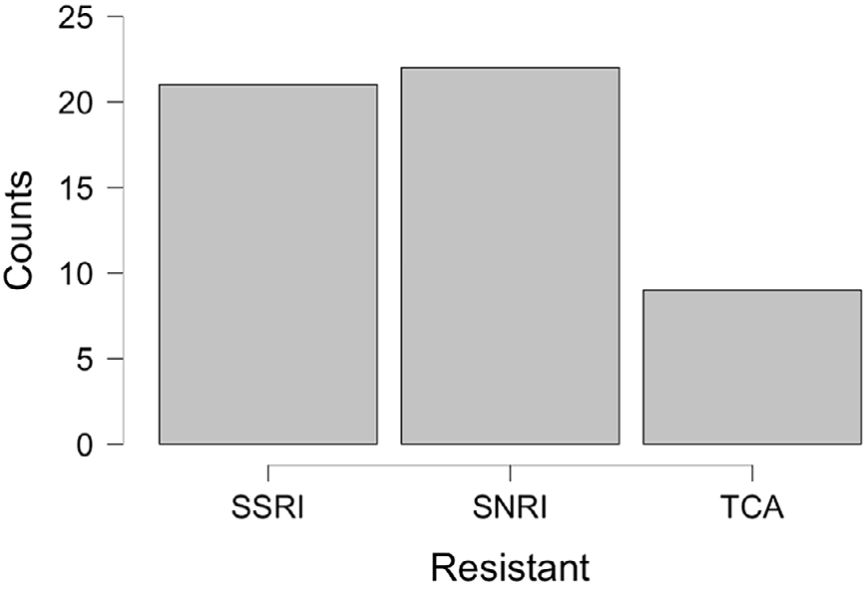

**Figure:**
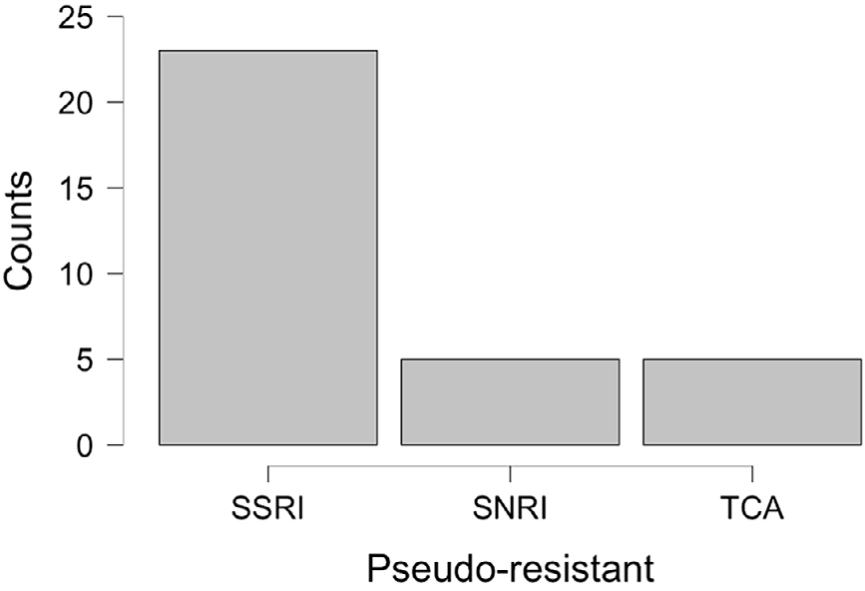

**Table:** 

Table 1				
	*Non-resistant (111)*	*Resistant (54)*	*Pseudo-resistant (35)*	*p-value*
Age	59.1±11.9	63.0±12.6	57.0±11.3	0.036*
Episodes of illness	3.8±2.1	4.0±1.9	3.0±1.8	0.036*
Personality disorders	27.0%	18.9%	48.6%	0.009**
Therapies:				0.014**
SSRI	62.4%	40.4%	69.7%	
SNRI	19.8%	42.3%	15.1%	
TCA	17.8%	17.3%	15.1%	
Augmentation	24.3%	38.9%	17.1%	0.05**
Remission	76.5%	59.5%	81.2%	CvsB:0.045** CvsA:0.587**

On the day of admission, non-responders were 44.5% of the sample, but 39.3% of them did not meet the *Resistant* criteria, defining the *Pseudo-resistant* group. *Pseudo-resistant* differed from others by younger age, fewer illness episodes, higher rate of personality disorders, and different therapies during hospitalization [Fig.1,2,3]. *Pseudo-resistant* remission rate, significantly greater than *Resistant one*, was comparable to *Non-resistant* [Tab.1]. *Kruskal-Wallis Test **Chi-Squared Test

**Conclusions:**

This study outlines a new group of depressed patients that, apparently drug-resistant, displays the same outcome as responders when treated with first-line drugs during hospitalization, certainly taking benefit from the psychoeducational program. Quick recognition of these patients could be crucial to giving optimal care.

**Disclosure:**

No significant relationships.

